# Determinants of breastfeeding initiation among mothers in Sydney, Australia: findings from a birth cohort study

**DOI:** 10.1186/s13006-017-0130-0

**Published:** 2017-09-15

**Authors:** Amit Arora, Narendar Manohar, Andrew Hayen, Sameer Bhole, John Eastwood, Steven Levy, Jane Anne Scott

**Affiliations:** 1 School of Science and Health, Western Sydney University, Campbelltown Campus, Penrith, NSW Australia; 2 Discipline of Paediatrics and Child Health, Sydney Medical School, Westmead, NSW Australia; 3 Oral Health Service, Sydney Local Health District and Sydney Dental Hospital, NSW Health, Surry Hills, Australia; 4 COHORTE Research Group, Ingham Institute of Applied Medical Research, Liverpool, NSW Australia; 5 Faculty of Health, University of Technology Sydney, Ultimo, NSW Australia; 6 Faculty of Dentistry, The University of Sydney, Surry Hills, NSW Australia; 7 Community Paediatrics, Sydney Local Health District, NSW Health, Croydon, Australia; 8 Department of Preventive and Community Dentistry, University of Iowa, Iowa City, USA; 9 School of Public Health, Curtin University, Perth, WA Australia

**Keywords:** Breastfeeding, initiation, Australia, Sydney, Cohort study

## Abstract

**Background:**

Breastfeeding has short-term and long-term benefits for both the infant and the mother. The objective of this study was to identify the incidence of breastfeeding initiation among women in South Western Sydney, and the factors associated with the initiation of breastfeeding.

**Methods:**

Child and Family Health Nurses recruited mother-infant dyads (*n* = 1035) to the *Healthy Smiles Healthy Kids* birth study in South Western Sydney, an ethnically and socio-economically diverse area, at the first post-natal home visit. A sample of 935 women completed a structured, interviewer-administered questionnaire at 8 weeks. Multivariate logistic regression analysis was used to identify those factors independently associated with the initiation of breastfeeding.

**Results:**

In total, 92% of women (*n* = 860) commenced breastfeeding in hospital. Women who completed a university degree were more likely to initiate breastfeeding compared to those who did not complete high school (AOR = 7.16, 95% CI 2.73, 18.79). Vietnamese women had lower odds of breastfeeding initiation compared to Australian born women (AOR = 0.34. 95% CI 0.13, 0.87). Women who had more than one child were less likely to breastfeed than those who had one child (AOR = 0.38, 95% CI 0.19, 0.79). Women who gave birth via a caesarean section were less likely to breastfeed their baby compared to those who had a vaginal delivery (AOR = 0.27, 95% CI 0.14, 0.52). Women who drank alcohol during pregnancy had 72% lower odds to breastfeed compared to those who did not drink alcohol during pregnancy (AOR = 0.28, 95% CI 0.11, 0.71). Women who reported that their partner preferred breastfeeding were more likely to initiate breastfeeding (AOR = 11.77, 95% CI 5.73, 24.15) and women who had chosen to breastfeed before pregnancy had more than 2.5 times the odds of breastfeeding their baby compared to those women who made their decision either during pregnancy or after labour (AOR = 2.80, 95% CI 1.31, 5.97).

**Conclusions:**

Women with lower levels of education, who consume alcohol during pregnancy, have more than one child, and make infant feeding decision after becoming pregnant, and those born in Vietnam should be targeted when implementing breastfeeding promotion programs. Further, women who deliver by caesarean section require additional breastfeeding support post-delivery and it is important to include fathers in breastfeeding related decisions and encourage them to participate in antenatal programs.

## Background

International recommendations including the World Health Organization (WHO) [1] and the Australian Dietary Guidelines [2] recommend infants should be exclusively breastfed for 6 months and continued further breastfeeding up to 12 months and beyond. These guidelines and recommendations are based on overwhelming evidence highlighting the short-term and the long-term benefits of breastfeeding for both the infant and the mother [3, 4]. Mothers are particularly encouraged to breastfeed as it provides infants with essential nutrients that are required for optimal growth and development and also enhances their immune system [3–5]. Breastfeeding protects against gastrointestinal and respiratory infections and otitis media in infancy, and reduces the risk of obesity and diabetes later in life [3, 4]. Breastfeeding also plays an important role in reducing infant mortality rates as well as biological and emotional well-being of infant and the mother [3–6].

Breastfeeding also provides benefits to the mother. Mothers who breastfeed are at a reduced risk for developing postmenopausal breast cancer, have higher bone density after menopause, experience a more timely and efficient return of the uterus to its pre-pregnancy state, and increased weight loss in the postpartum period [3–7]. Breastfeeding mothers report reduced stress levels, which may be caused by increased prolactin levels [7]. Women who breastfeed have an increased length of time between pregnancies, a decreased risk of ovarian cancer, a decreased risk of postmenopausal hip fracture, and a reduced risk to type 2 diabetes mellitus [3–7].

Undertaking research on breastfeeding practices is crucial to identify specific population sub-groups of women who decide not to breastfeed and the reasons for not complying with international recommendations. Although a range of factors that influence breastfeeding initiation have been reported in the literature [8], these factors differ across cultures, populations, regions, and countries [9–12]. In Australia, the incidence of breastfeeding is high (96%) [13], however, the rates vary across ethnic and socio-economic groups. For example, the Perth Infant Feeding Study noted that Australian-born mothers had significantly higher breastfeeding initiation rates in comparison to those born outside Australia [14]. Further, Amir and Donath investigated the changes in breastfeeding initiation rates across three national surveys in Australia and concluded that although breastfeeding initiation rates increased from 86.0% in 1995 to 88.0% in 2004–05 [15], they were consistently lower for women from lower socio-economic backgrounds [15].

South Western Sydney is an ethnically diverse region in New South Wales (NSW), Australia with high levels of social disadvantage [16]. The data related to predictors of breastfeeding initiation in NSW, particularly in South Western Sydney, are limited [17]. For these reasons, it is important to investigate breastfeeding initiation and its associated factors in this region to help to identify strategies to promote and support breastfeeding initiation and to identify women most at risk of not breastfeeding. The aims of this study therefore, were to identify the incidence and determinants of breastfeeding initiation in one of Australia’s most culturally and linguistically diverse communities and to report on the main reasons why mothers do not initiate breastfeeding.

## Methods

### Study background

This paper analyses data obtained from an ongoing birth cohort study, ‘Healthy Smiles Healthy Kids’, investigating the relationship between early childhood feeding patterns, oral health and obesity among children in South Western Sydney [18]. Women who gave birth to a live infant with no health condition between October 2009 and February 2010 in public hospitals located under the catchment of the former South Western Sydney Area Health Service (now classified as Sydney and South Western Sydney Local Health Districts) were approached to be a part of this study. Child and Family Health Nurses (CFHNs) recruited mother-infant dyads at the first post-natal home visit at 4 to 6 weeks, as this is the primary point of community-based health professional contact for newborn children and their carers/parents [19, 20]. At the first post-natal visit, CFHNs explained the project to the mothers and obtained a written informed consent. If requested, the nurses were able to arrange for interpreter services for non-English speaking parents and language appropriate written materials were provided for the major ethnic groups living in this region (i.e. Vietnamese, Arabic, Assyrian, Cambodian, Cantonese, Hindi, Mandarin, and Samoan).

### Data collection

Basic demographic information and infant feeding information were collected via a telephone interview conducted when the child was 8 weeks old. The questionnaire used in this study was adapted from the first and second Perth Infant Feeding studies [21, 22], to determine if breastfeeding had been initiated and identify the factors known or suspected to be linked with breastfeeding initiation.

### Outcome variable

Initiation of breastfeeding is defined as a woman having breastfed her child, either from the breast or expressed breast milk, on at least one occasion.

### Explanatory variables

A number of independent variables (socio-demographic, biomedical and psychosocial) identified in the literature as being identified as being associated with breastfeeding initiation were investigated (Table 2). ‘Mothers age’ was recorded as a continuous variable and then categorised into groups (<25 years, 25–29 years, 30–34 years, >35 years). Mothers were asked an open-ended question about their ‘occupation’ and these were classified as per the Census occupation codes (Home duties, unskilled, sales/clerical, managers, and professionals). Socio-economic status was defined using the residential postcode which was classified as per the Index of Relative Socioeconomic Advantage and Disadvantage (IRSAD), one of the Socio-economic Indexes for Areas developed by the Australian Bureau of Statistics [23]. ‘Birthweight’ was recorded as a continuous variable and then categorised into ‘low’ (<2500 g) and ‘average or greater’ (≥2500 g). ‘Parity’ was recorded as a continuous variable and later categorised as ‘primiparous’ and multiparous’. The behaviours ‘smoking during pregnancy’ and ‘alcohol during pregnancy’ were recorded as a ‘yes’ or a ‘no’.

### Statistical analysis

Data were entered and analysed using the Statistical Package for Social Sciences, Version 22 (SPSS for Windows, SPSS Inc., Chicago, IL, USA). Univariate logistic regression analysis was performed followed by multivariate logistic regression analysis to determine which individual variables could best predict the initiation of breastfeeding. The full model was reduced using the backward stepwise procedure and the fitness of model was assessed at every step to avoid dropping non-significant variables that affected the model fitness. All variables in the final model were variables from which, when excluded, the change in deviance compared with the corresponding *Χ* ^2^ test statistic on the relevant degrees of freedom was significant (*p* < 0.05).

## Results

A total of 1500 mothers were invited to participate in this study and 1035 mothers agreed to participate (69% consent). All the mothers who declined to participate (*n* = 465) were asked three short questions related to their socio-demographic status and chosen method of feeding to allow comparison with the respondents (*n* = 1035). There were no significant differences between the two groups with respect to age (*X* ^2^ *=* 4.75, *p* = 0.153), level of education (*X* ^2^ *=* 6.65, *p* = 0.328) and chosen method of feeding (*X* ^2^ *=* 2.46, *p* = 0.813). These findings suggest that the sample was representative of the population from which it was drawn. At the time of the first interview, 100 women either declined to continue with the cohort study or were not contactable after 7 phone attempts on weekdays and/or weekends. While 69% (n = 1035) consented to participate in the study, results were obtained from 62.3% (*n* = 935). Table 1 shows the socio-demographic and the biomedical characteristics of the study participants. Of the 935 mothers who completed the baseline interview, 860 women (92%) initiated breastfeeding.

**Table 1:** Sociodemographic and biomedical characteristics of the participants (*n* = 935)

Characteristic	Number	Percent
Sociodemographic factors		
Maternal age (years)		
< 25	93	9.9
25–29	255	27.3
30–34	351	37.5
≥ 35	236	25.2
Maternal education		
< Year 12	141	15.1
Year 12 completed	208	22.2
College/TAFE	179	19.1
University	407	43.5
Marital status		
Married	734	78.5
Living with a partner/ De facto	110	11.8
Single Mother	91	9.7
Mother’s occupation		
Home duties/ student	170	18.2
Managers	61	6.5
Professionals	241	25.8
Sales/Clerical	294	31.4
Unskilled	169	18.1
Mother’s country of birth		
Australia	436	46.6
China	57	6.1
Vietnam	132	14.1
Asia other	111	11.9
Middle East/Africa	81	8.7
Others	117	12.5
Partner’s country of birth		
Australia	359	38.4
China	42	4.5
Vietnam	115	12.3
Asia Other	105	11.2
Middle East/Africa	96	10.3
Others	120	12.8
Index for relative socioeconomic disadvantage		
Least Disadvantaged	50	5.3
2nd Quintile	238	25.5
3rd Quintile	80	8.6
4th Quintile	183	19.6
Most Disadvantaged	384	41.1
Biomedical factors		
Parity		
Primiparous	468	50.1
Multiparous	467	49.9
Infant gender		
Female	458	49.0
Male	477	51.0
Infant birth weight		
Average or greater	889	95.1
Low	46	4.9
Smoking status of the mother post partum		
No	852	91.1
Yes	83	8.9
Smoking status of the mother during pregnancy		
No	884	94.5
Yes	51	5.5
Alcohol drinking status of the mother post partum		
No	633	67.7
Yes	302	32.3
Alcohol drinking status of the mother during pregnancy		
No	843	90.2
Yes	92	9.8
Method of delivery		
Vaginal	653	69.8
Caesarean	282	30.2

### Univariate analysis

Table 2 lists the socio-demographic, biomedical, and psychosocial factors known or suspected to be associated with breastfeeding initiation. Higher levels of maternal education and maternal occupational status were associated with increased odds for breastfeeding initiation, whilst being a single mother was associated with decreased odds of not initiating breastfeeding. Furthermore, women from some Asian countries (eg: Indian sub-continent) had higher odds of breastfeeding initiation compared to Australian born women. Mothers who had more than one child, or who smoked cigarettes during pregnancy, or drank alcohol during pregnancy, or who had a caesarean delivery were less likely to initiate breastfeeding. Women who had chosen their preferred feeding method before pregnancy were more likely to breastfeed than women who had made their decision either during pregnancy or after labour. There was also a strong association between the initiation of breastfeeding and if the partner preferred breastfeeding.

**Table 2:** Association between socio-demographic, biomedical, and psychosocial factors and breastfeeding initiation (n = 935)

Variable	Breastfeeding Initiation^a^		Univariate odds ratio^b^	
	Yes	No		
	N (%)	N (%)	OR	95%CI
Sociodemographic factors				
Maternal age (years)				
< 25	84 (90.3)	9 (9.7)	1.00	
25–29	235 (92.2)	20 (7.8)	1.26	0.55, 2.87
30–34	323 (92.0)	28 (8.0)	1.24	0.56, 2.72
≥ 35	218 (92.4)	18 (7.6)	1.30	0.56, 3.00
Maternal education				
< Year 12	114 (80.9)	27 (19.1)	1.00	
Year 12 completed	182 (87.5)	26 (12.5)	1.66	0.92, 2.98
College/TAFE	169 (94.4)	10 (5.6)	4.00	1.86, 8.59**
University	395 (97.1)	12 (2.9)	7.80	3.83, 15.88**
Marital status				
Married	689 (93.9)	45 (6.1)	1.00	
Living with a partner/ De facto	98 (89.1)	12 (10.9)	0.53	0.27, 1.04
Single mother	73 (80.2)	18 (19.8)	0.26	0.15, 0.48**
Mother’s occupation				
Home duties/ student	148 (87.1)	22 (12.9)	1.00	
Unskilled	152 (89.9)	17 (10.1)	1.33	0.68, 2.60
Sales/Clerical	268 (91.2)	26 (8.8)	1.53	0.84, 2.80
Managers	58 (95.1)	3 (4.9)	2.87	0.83, 9.97
Professionals	234 (97.1)	7 (2.9)	4.97	2.07, 11.92**
Mother’s country of birth				
Australia	398 (91.3)	38 (8.7)	1.00	
China	55 (96.5)	2 (3.5)	2.63	0.62, 11.19
Vietnam	117 (88.6)	15 (11.4)	0.74	0.40, 1.40
Asia other	109 (98.2)	2 (1.8)	5.20	1.24, 21.91*
Middle East/Africa	77 (95.1)	4 (4.9)	1.84	0.64, 5.30
Others	103 (88.0)	14 (12.0)	0.70	0.37, 1.34
Partner’s country of birth				
Australia	327 (91.1)	32 (8.9)	1.00	
China	41 (97.6)	1 (2.4)	4.01	0.53, 30.14
Vietnam	103 (89.6)	12 (10.4)	0.84	0.42, 1.69
Asia other	101 (96.2)	4 (3.8)	2.47	0.85, 7.15
Middle East/Africa	93 (96.9)	3 (3.1)	3.03	0.91, 10.13
Others	110 (91.7)	10 (8.3)	1.08	0.51, 2.26
Mother intended to be employed/study at 6 months post-partum				
No/Undecided	672 (91.4)	63 (8.6)	1.00	
Yes	188 (94.0)	12 (6.0)	1.47	0.78, 2.78
Index for relative socioeconomic disadvantage				
Least disadvantaged	48 (96.0)	2 (4.0)	1.00	
2nd quintile	223 (93.7)	15 (6.3)	0.62	0.14, 2.80
3rd quintile	77 (96.3)	3 (3.8)	1.07	0.17, 6.63
4th quintile	162 (88.5)	21 (11.5)	0.32	0.07, 1.42
Most disadvantaged	350 (91.1)	34 (8.9)	0.43	0.10, 1.84
Biomedical factors				
Parity				
Primiparous	444 (94.9)	24 (5.1)	1.00	
Multiparous	416 (89.1)	51 (10.9)	0.44	0.27, 0.73*
Infant gender				
Female	421 (91.9)	37 (8.1)	1.00	
Male	439 (92.0)	38 (8.0)	1.02	0.63, 1.63
Infant birth weight				
Average or greater (≥ 2500 g)	819 (92.1)	70 (7.9)	1.00	
Low (<2500 g)	41 (89.1)	5 (10.9)	0.70	0.27, 1.83
Method of delivery				
Vaginal	618 (94.6)	35 (5.4)	1.00	
Caesarean	242 (85.8)	40 (14.2)	0.34	0.21, 0.55**
Smoking status of the mother during pregnancy				
No	821 (92.9)	63 (7.1)	1.00	
Yes	39 (76.5)	12 (23.5)	0.25	0.12, 0.50**
Alcohol drinking status of the mother during pregnancy				
No	785 (93.1)	58 (6.9)	1.00	
Yes	75 (81.5)	17 (18.5)	0.33	0.18, 0.59**
Psychosocial factors				
Partner prefers breastfeeding				
No or ambivalent	204 (82.3)	44 (17.7)	1.00	
Yes	562 (97.7)	13 (2.3)	9.32	4.92, 17.67**
Infant feeding decision made before pregnancy				
No	128 (87.1)	19 (12.9)	1.00	
Yes	732 (92.9)	56 (7.1)	1.94	1.12, 3.37*

*OR* Odds ratio, *95%CI* 95% confidence interval

^a^ The total of the categories do not always add up to 935 due to missing or incomplete data for some items

^b^ The univariate odds ratio indicates the likelihood of being breastfed ever

* *p* < 0.05 ** *p* < 0.001

### Multivariate analysis

Multivariate logistic regression analysis was used to determine which variables could best explain the chosen method of feeding. Those factors that were independently associated with initiation of breastfeeding are presented in Table 3. After potential confounding factors were controlled for, level of maternal education was significantly associated with breastfeeding initiation. Women who completed a university degree were more likely to initiate breastfeeding compared to those who did not complete high school (AOR = 7.16, 95% CI 2.73, 18.79). Vietnamese women had lower odds of breastfeeding initiation compared to Australian born women (AOR = 0.34. 95% CI 0.13, 0.87). Women who had more than one child were less likely to breastfeed than those who had one child (AOR = 0.38, 95% CI 0.19, 0.79). Women who gave birth via a caesarean section were less likely to breastfeed their baby compared to those who had a vaginal delivery (AOR = 0.27, 95% CI 0.14, 0.52). Women who drank alcohol during pregnancy had 72% lower odds to breastfeed compared to those who did not (AOR = 0.28, 95% CI 0.11, 0.71). Women who reported that their partner preferred breastfeeding were more likely to initiate breastfeeding (AOR = 11.77, 95% CI 5.73, 24.15) and women who had chosen to breastfeed before pregnancy had more than 2.5 times the odds of breastfeeding their baby compared to those women who made their decision either during pregnancy or after labour (AOR = 2.80, 95% CI 1.31, 5.97).

**Table 3:** Factors independently^a^ associated with initiation of breastfeeding after adjustment for potential confounders^b^ (*n* = 822)

Variable		Ever initiated breastfeeding	
	AdjOR^c^	95%CI	*p*-value
Sociodemographic factors			
Maternal education			
< Year 12	1.00		
Year 12 completed	1.36	0.56, 3.31	0.498
College/TAFE	2.28	0.84, 6.16	0.105
University	7.16	2.73, 18.79	<0.001
Mother’s country of birth			
Australia	1.00		
China	0.75	0.15, 3.74	0.729
Vietnam	0.34	0.13, 0.87	0.025
Asia other	1.93	0.41, 9.09	0.406
Middle East/Africa	1.86	0.51, 6.81	0.348
Others	0.41	0.17, 1.00	0.050
Biomedical factors			
Parity			
Primiparous	1.00		
Multiparous	0.38	0.19, 0.79	0.010
Method of delivery			
Vaginal	1.00		
Caesarean	0.27	0.14, 0.52	<0.001
Alcohol drinking status of the mother during pregnancy			
No	1.00		
Yes	0.28	0.11, 0.71	0.008
Psychosocial factors			
Partner prefers breastfeeding			
No or ambivalent	1.00		
Yes	11.77	5.73, 24.15	<0.001
Infant feeding decision made before pregnancy			
No	1.00		
Yes	2.80	1.31, 5.97	0.008

^a^ All variables in the final model were variables for which, when excluded, the change in deviance compared with the corresponding χ^2^ test statistic

on the relevant degrees of freedom was significant

^b^ Adjusted for maternal age, marital status, maternal occupation, father’s country of birth, mother’s future employment intentions,

index of relative socioeconomic disadvantage, infant gender, infant birth weight, smoking status of the mother during pregnancy

^c^ *AdjOR* Adjusted Odds Ratio, *95%CI* 95% confidence interval

## Discussion

This study provides an insight into the socio-demographic, biomedical and psychosocial factors associated with breastfeeding initiation practices of mothers residing in South Western Sydney. The independent predictors included factors such as maternal education, maternal alcohol consumption during pregnancy, giving birth via a caesarean section, partner support for breastfeeding, and timing of infant feeding decision. In the present study, nine out of 10 of women commenced breastfeeding indicating strong adherence to WHO infant feeding guidelines [1]. This is in accordance with previous local and national reports [13, 22, 24, 25].

Maternal socio-demographic factors such as age and education that might influence early childhood feeding are non-modifiable and as such are not subject to direct clinical intervention. Still, they can predict which mothers may be less likely to initiate and continue breastfeeding, thus identifying sub-groups that would benefit the most from health promotion strategies. Maternal age has consistently been associated with breastfeeding initiation [26, 27]; in general, older mothers are more likely to initiate breastfeeding and to continue breastfeeding for longer [28]. Conversely in this study, after controlling for covariates and potentially confounding variables, maternal age was not found to be associated with breastfeeding initiation. Similar findings have also been observed in earlier Australian research which adjusted for similar counfounders [21, 29]. This finding, is not unexpected in this study given that the cohort was relatively mature with only 10% of subjects being aged less than 25 years and because the initiation of breastfeeding was near universal [29]. The present study endorses the existing evidence that level of maternal education amongst Western women could independently predict breastfeeding initiation. Similar results have been reported by recent Australian studies [26, 27, 30] and a recent cohort study in the United States [31]. In this study, women born in Vietnam were less likely to initiate breastfeeding compared to Australian born mothers (Table 3). Other researchers have also reported findings that Vietnamese migrant women are less likely to breastfeed than Australian born women [25, 32] and the proportion of Vietnamese mothers in this study initiating breastfeeding was 10% lower than that reported for Vietnamese women in their home country [33]. Reasons previously cited for low breastfeeding rates among Vietnamese migrant women are cultural beliefs related to colostrum [25], inconvenience [32], perceptions of impaired quality of milk [34], economic reasons such as the need to return to work [31, 34], a decrease in social support [32, 35], perception that more affluent families do not breastfeed their own babies [35], and a desire to conform to the perceived cultural norm of the new country [34]. In this study, reasons for breastfeeding initiation were not explored in-depth and therefore it is difficult to draw conclusions for the lower rates of initiation.

Similar to prior studies [31, 36] a positive association was found between parity and breastfeeding initiation among the target population. In this study, multiparous women were less likely to initiate breastfeeding compared to their primparous counterparts. We speculate that multiparous women who previously experienced breastfeeding difficulties did not initiate breastfeeding. A recent study from the United States [37] reported a notable decrease in breastfeeding initiation with increasing birth order: compared to the first birth, the odds for non-initiation after a second delivery almost doubled (OR = 1.83, 95%CI1.42, 2.35) and the odds for non-initiation after a third delivery were further increased (OR = 2.44, 95%CI 1.56, 3.82). The public health implication of this observation in our study is that breastfeeding rates among multiparous women might be improved through targeted pre- and post-natal support of women with a history of unsuccessful breastfeeding.

Also, consistent with the literature [38], women who consumed alcohol during pregnancy were less likely to initiate breastfeeding. It is not clear if there is a biological mechanism for this association or whether mothers who drank chose not to breastfeed out of concern for the safety of their child or whether this association reflects a clustering of unhealthy lifestyle practices [38]. Nevertheless, we recommend that primary care providers advise pregnant women about the harmful effects of alcohol on breastfeeding success and refer women to where they can find information alcohol consumption and breastfeeding [39]. Interestingly, there was no association with mothers’ smoking status during pregnancy and breastfeeding initiation in the current study; which is in contrast to the findings of an earlier study in South Western Sydney [40]. While a review of epidemiological studies on the association between maternal smoking and breastfeeding found that in general, women who smoke are less likely to intend to breastfeed and to initiate breastfeeding, this association has not been consistently reported in Australian studies [29]. It has been suggested that the reason for this association is more likely to be psychosocial rather than biological [41]. The above findings can assist to identify disadvantaged target populations to effectively implement public health interventions for promoting breastfeeding initiation.

Caesarean section is a significant barrier to the initiation of breastfeeding. Similar to other international research [42, 43], a negative association was observed between caesarean section and breastfeeding initiation in the current study. Delayed skin-to-skin contact [44], prolonged mother-infant separation, an increased length of stay in hospital, as well as maternal endocrinological changes induced by surgery have all been postulated as reasons for failure to initiate breastfeeding in mothers who undergo a caesarean section [43]. Since caesarean section is an increasingly common method of delivery [45], it is important that these women are provided additional breastfeeding support post-delivery.

Although in this study, marital status was not found to be associated with breastfeeding initiation, the father’s preference for breastfeeding was reported to be a strong positive factor associated with the initiation of breastfeeding. The results of this study support and strengthen the findings of previous studies [22, 29]. The degree to which a woman’s partner will influence her decision to breastfeed will vary according to woman’s age, social class and cultural background [46]. For instance, Anglo-American women identified their husband as being their major source of support regarding infant feeding decision [12]. Studies of women from Indian sub-continent highlighted the importance of mothers and grandmothers on infant feeding decisions and providing practical support in breastfeeding [47, 48]. In this study, fathers were not interviewed, and answers to paternal preferences represent mother’s opinion about the husband’s attitude. Nevertheless, the results of this research indicate the need to include fathers in breastfeeding related decisions and to participate in antenatal programs where breastfeeding is discussed. Providing fathers from ethnically diverse backgrounds with culturally appropriate breastfeeding information to become breastfeeding advocates will likely increase breastfeeding practices in Sydney, Australia.

Another psychosocial factor found to be a predictor of breastfeeding initiation was the timing when the infant feeding decision was made. In this study, the majority of the women had made their infant feeding decision before they conceived. There was an association between when the decision was made with the initiation of breastfeeding. Women who chose their feeding method before becoming pregnant were more likely to breastfeed than women who made their decision after becoming pregnant (Table 3). This positive correlation is supported by previous research [29]. The time at which a woman makes her feeding decision may be viewed as a marker of maternal commitment. Women who decide to breastfeed prior to becoming pregnancy are likely to have a stronger desire and determination to breastfeed than women who do not consider infant feeding until later in their pregnancy.

This study has a number of limitations. First, the outcome was measured based on self-report which may have led to a recall bias that may have underestimated or overestimated the association between breastfeeding initiation and key study factors. However, it is worth noting that the recall period for this study was short. Second, in for some explanatory variables (e.g. country of birth and IRSAD), the number of women in some categories was small (<5). Given the small proportion of women who did not initiate breastfeeding this resulted in a rare events bias reflected in the large confidence intervals around the odds ratio [49]. A larger sample of women from countries such as China, Middle East/Africa and other parts of Asia would have provided more statistically robust findings and these findings should be interpreted with care. Third, this study only reports on breastfeeding initiation in South Western Sydney; therefore the findings may not be generalisable to all of New South Wales or Australia. Nevertheless, this study is one of the few studies that report on breastfeeding initiation in South Western Sydney, one of the most ethnically diverse area in Australia with high levels of social disadvantage. Hence, the results may be used to inform both local and national nutrition programs as the findings provide useful insights into those population groups at risk of not initiating breastfeeding, as well as potentially modifiable risk factors for this practice.

## Conclusions

This study provides further insight into breastfeeding initiation practice in one of Australia’s most ethnically diverse areas with high levels of social disadvantage. While breastfeeding initiation rates in South Western Sydney are high, there are some population groups nevertheless which are less likely to initiate breastfeeding than Australian mothers. Those women with lower levels of education, who consume alcohol during pregnancy, have more than one child, make their infant feeding decision after becoming pregnant, and those of Vietnamese ethnicity should be targeted when implementing breastfeeding promotion programs. Furthermore, women who deliver by caesarean section require additional breastfeeding support post-delivery and it is important to include fathers in breastfeeding related decisions and encourage them to participate in antenatal programs.

## Abbreviations

*X*^2^: Chi-square95%CI 95% confidence interval
AOR: Adjusted Odds Ratio
BFHI: Baby Friendly Hospital Initiative
CFHN: Child and Family Health Nurses
NSW: New South Wales
UNICEF: United Nations International Children’s Emergency Fund
WHO: World Health Organization
